# Research, adapt, and overcome: Changes in submission rates to endocrinology scientific journals during the COVID-19 pandemic

**DOI:** 10.3389/fendo.2022.998269

**Published:** 2022-08-31

**Authors:** Dror Ben Ephraim Noyman, Mogher Khamaisi

**Affiliations:** ^1^ Department of Ophthalmology, Rambam Health Care Campus, Haifa, Israel; ^2^ Department of Internal Medicine D and Institute of Endocrinology, Rambam Health Care Campus, Haifa, Israel; ^3^ Ruth & Bruce Rappaport Faculty of Medicine, Technion-IIT, Haifa, Israel

**Keywords:** COVID, endocrinology, research activity, submissions rates, scientific journals

## Introduction

The novel corona virus pandemic of COVID-19 has initiated in Wuhan, China at the end of 2019, and has quickly spread thereafter to the majority of the world by the beginning of 2020, affecting every level of our lives. Soon, governments imposed heavy restrictions, travel bans, quarantines, and even lockdowns, fluctuating in severity according to local policies, new variants, and waves of viral infections. In-turn, these have caused many people to remain at home or in close proximity for long periods of time, transferring many activities such as educational and investigational activities to the online domain, refraining from physical human contact and on-site collaborations (1). For investigators and clinicians, the consequences in many cases were inevitable suspension of clinical, preclinical, and any other trials requiring on-site presence, and at times even cancellations of such studies (1–3). Moreover, the shifting of efforts between different types of studies and even clinical activity was forced (1, 2, 4, 5). Likewise, clinical activity itself was altered in character and amount, as the pandemic presented the medical community with new, unmet challenges. However, the situation created a unique opportunity at times, both for investigation of a singular event in the medical field, and for advancing work on raw manuscripts and unrefined existing data.

While the pandemic’s influence on the medical field is still with us, it has certainly tuned down, and as its’ “acute phase” is fading, seems a good time for reckoning among the medical research community as to its behavior during these radical times. Given that nearly every investigator would be glad to have their study published- in our current work, we have looked at the number of manuscripts submitted to scientific journals, as a measure of the pandemics impact on the volume and character of investigational activity.

## Methods

We have contacted the editorial and publication offices of 235 known Endocrinology scientific journals in request for the number and type of submissions to their esteemed journals during the two years prior to the pandemic and the two years during the pandemic. Twenty-nine journals’ editors and publishers have generously agreed to contribute their data, which is summarized in the table below (**Table 1**). According to the publishers’ and editors’ request, journals’ titles are not stated to assure confidentiality of their identity. Impact Factors (IF) for each journal are not shown for the same reason, as well as for the reporting of it being variable. Instead, normalized, and multiplied by a constant, SCImago Journal Rank (SJR) (6) indexes are reported, for preserving the confidentiality of the journals while allowing the readers an orientated observation, as its reporting is coherent and it too serves as a measure of scientific influence of journals.

**Table 1:** Summary of submission and publication data of every contributing journal by 2 year time intervals.

	Submission Type								Total Published		Factored SJR	
	Total		Reviews		Research		Case Reports					
**Year/Journal**	**2018-2019**	**2020-2021**	**2018-2019**	**2020-2021**	**2018-2019**	**2020-2021**	**2018-2019**	**2020-2021**	**2018-2019**	**2020-2021**	**2018**	**2021**
**1**	3346	3963	110	123	3101	3653	n.a	n.a	529	554	0.850	1.000
**2**	1642	1587	28	45	1598	1529	n.a	n.a	500	403	0.571	0.389
**3**	773	1111	31	39	647	822	54	111	366	328	0.676	0.477
**4**	2434	2315	109	131	2099	1808	n.a	n.a	555	604	0.376	0.550
**5**	1912	1678	n.a	n.a	n.a	n.a	n.a	n.a	696	553	0.600	0.476
**6**	2391	2654	n.a	n.a	n.a	n.a	n.a	n.a	690	785	0.456	0.308
**7**	1256	1122	n.a	n.a	n.a	n.a	n.a	n.a	276	220	0.416	0.287
**8**	X	1.5*X	n.a	n.a	n.a	n.a	n.a	n.a	299	450	0.355	0.287
**9**	1180	938	n.a	n.a	n.a	n.a	n.a	n.a	501	298	0.391	0.304
**10**	659	2522	90	427	520	2016	2	5	396	1364	0.274	0.271
**11**	3730	1370	n.a	n.a	n.a	n.a	n.a	n.a	288	359	0.316	0.300
**12**	1033	1504	n.a	n.a	n.a	n.a	n.a	n.a	257	361	0.242	0.204
**13**	1836	1881	70	71	1611	1659	n.a	n.a	393	386	0.341	0.216
**14**	379	511	n.a	n.a	n.a	n.a	n.a	n.a	105	160	0.253	0.166
**15**	360	342	n.a	n.a	n.a	n.a	n.a	n.a	104	61	0.362	0.183
**16**	1050	1328	34	46	926	1160	n.a	n.a	266	288	0.208	0.204
**17**	1417	1919	137	231	1140	1472	38	64	113	187	0.235	0.245
**18**	209	196	n.a	n.a	n.a	n.a	n.a	n.a	93	81	0.226	0.132
**19**	231	463	n.a	n.a	n.a	n.a	n.a	n.a	70	133	0.187	0.138
**20**	55	102	12	19	23	50	4	6	40	69	n.a	0.198
**21**	335	456	n.a	n.a	n.a	n.a	n.a	n.a	128	147	0.124	0.074
**22**	75	107	12	20	15	40	n.a	n.a	103	120	0.052	0.062
**23**	98	66	10	17	43	65	n.a	n.a	83	79	0.044	0.037
**24**	1504	1639	n.a	n.a	n.a	n.a	n.a	n.a	587	525	0.386	0.295
**25**	1037	1090	n.a	n.a	n.a	n.a	n.a	n.a	295	291	0.251	0.247
**26**	332	261	n.a	n.a	n.a	n.a	n.a	n.a	128	126	0.232	0.206
**27**	217	252	n.a	n.a	n.a	n.a	n.a	n.a	124	97	0.258	0.226
**28**	1006	898	n.a	n.a	n.a	n.a	n.a	n.a	580	352	0.257	0.200
**29**	572	708	n.a	n.a	n.a	n.a	n.a	n.a	320	208	0.182	0.142
**Total**	**29157**	**31305**	**643**	**1169**	**11723**	**14274**	**98**	**186**	**8189**	**9036**	**Not applicable**	
**Total** **Increase [%]**	**6.2%**		**81.8%**		**21.8%**		**89.8%**		**7.9%**	
**Average Increase [%]**	**23.4%**		**72.1%**		**64.3%**		**93.5%**		**14.7%**		**-16.6%**	

Where supplied- a breakdown of the submissions into Review, Research, and Case Report type is displayed. The third and fourth columns to the right depict the number of papers eventually published during that time frame in the corresponding journal. The two columns to the right represent normalized and factored SJR scores for the years 2018 (second to the right) and 2021 (first to the right). The summary rows describe the total number of papers in the category, the total increase between the two timeframes within that category, and the average increase per journal between the two timeframes within that category.

Missing data: n.a, not available; X, absolute number not supplied.

Calculations of total sums and changes in bold.

## Results

Compared to the two years prior, the total number of submissions during the two years of the pandemic increased by 6.2%. However, the average increase per journal was 23.4%, ranging from a 63.3% decrease to a 282.7% increase. Out of 29 journals, 18 (62.1%) have experienced an increase in the total number of submissions. No correlation was found between the SJR scores for 2018 and 2021, and the change in submission rate, where the Pearson correlation coefficients were -0.05 and -0.15 with respect. The changes in SJR scores through this period of time (from 2018 to 2021) had a non-significant correlation to the change in submission rates as well with a coefficient of 0.22.

Only 11 journals supplied a breakdown of the types of submissions. However, all of the above had an increase in submissions of reviews with a total of 81.8% increase altogether (72.1% average increase per journal). Only 9 out of 11 journals experienced an increase in submission of research manuscripts, with a total increase of 21.8%, and an average of 64.3% increase per journal. Four out of four journals which reported a breakdown for manuscripts of case reports experienced an increase, and the total increase was of 89.8% (with an average of 93.5% per journal).

While there was a total of 7.9% increase in publications with a 14.7% average per journal, only 8 (27.6%) journals have increased their acceptance rate. Similarly to the change in submission, no correlation was found in this case between the SJR scores of 2018 and 2021 and the change in acceptance rate, nor did the changes in SJR scores along the stated years, where the Pearson correlation coefficients were -0.02, -0.05, and 0.07 with respect.

The journals’ SJR score was reduced by an average of 16.6% through the examined period of time, ranging between a 49.4% reduction to 46.3% increase. Between the initial SJR score of 2018 and the change in SJR score there was a higher correlation with a coefficient of 0.41.

## Discussion

A small number of the addressed journals have agreed to cooperate and contribute, thus limiting the power of conclusion that can be drawn from this analysis. However, the ratio of response was not significantly different than other analyses done in this manner in other fields (7).

Interestingly, the field of endocrinology experienced a smaller increase in the total influx of submissions compared to a 42.5% increase described previously in Ophthalmology (7). This may be a result of the fundamental differences between the nature of work between the two fields. While eye clinics have plenty of elective activity, which was likely postponed many times in lockdowns, enabling the clinician to attend to research activity; endocrinologists are part of the internal medicine realm, and as such were put often in the front-line of treating COVID-19 patients. Moreover, with the rise of telehealth capabilities and implementation, clinical endocrinologists could partially manage established patients such as in the case of diabetes (8, 9) and were most likely somewhat occupied by such activity.

Only partial data was available regarding the types of submissions. However, the unanimous increase in reviews, emphasizes the effect of the pandemic in shifting investigational activity to types of studies which do not require human contact and can be done remotely. While this may be attributed to the rising technological advancement in telemedicine which is likely to direct investigational activity to studies achievable remotely (10), it is hard to determine its exact effect as too few studies were published on the subject. Yet, the greatest growth of all submission types during the pandemic was in case reports. These indicate the persistence of clinical activity, as they are derived from it, and may be a result of the urge to promptly report the vast endocrinological effect of COVID-19, which was being discovered at the time, and included phenotypes of thyroid disorders (11), metabolic disturbances in diabetes control (9), and even Addison-like presentations.

As for community standards- although there was an increase in publications, only the minority of journals had increased acceptance rate, and the average acceptance rate per journal has decreased, giving evidence to the preservation of research quality control and the journals’ respect for the level of information publicly published, keeping the same quality evaluation standards and strictness even at radical times. In accordance with that, and with similar findings in the field of ophthalmology, editors should be aware for the need for more reviewers to accomplish uncompromised publication both quality and timely-wise.

The only notable correlation was the positive one found between the initial SJR score of 2018 and the change in SJR score between the years. While it may indicate a slight rally of the more established journals getting stronger, it should be acknowledged that it, too, did not produce a meaningful enough coefficient to draw powerful conclusions.

It is worth noting that as a whole, the average SJR score was significantly reduced. This needs to be pondered upon with respect to the circumstances, as the SJR, unlike the IF, gives weight to the prestige of the citing journal (6). Thus, it may be speculated, that while the reduction could be the result of less citation, it could also be due to the researchers’ more segregated approach through the pandemic or the shifting of investigational activity to types of research, as discussed above, which tend to be less cited in the short term. Several reasons could be postulated on this matter, either way, comparing the change in SJR scores in the field of endocrinology to that of other fields, should more analyses like this be carried out- should provide interesting insights.

Although the pandemic imposed great changes on endocrinologists both as clinicians and researchers, it seems the endocrinological community has managed not only to effectively aid in the public effort, but also it did not neglect the need for knowledge preservation as it harnessed the pandemic’s downsides and restrictions and turned them into efficient research activity time to supply the future community with quality education, all without compromise of the quality of information.

## Author contributions

DN contributed to the conception of the study. DN and MK contributed to the design of the study. DN organized the database and performed the statistical analysis and wrote the first draft of the manuscript. MK provided guidance and wrote sections of the manuscript. All authors contributed to manuscript revision, read, and approved the submitted version.

## Conflict of interest

The authors declare that the research was conducted in the absence of any commercial or financial relationships that could be construed as a potential conflict of interest.

## Publisher’s note

All claims expressed in this article are solely those of the authors and do not necessarily represent those of their affiliated organizations, or those of the publisher, the editors and the reviewers. Any product that may be evaluated in this article, or claim that may be made by its manufacturer, is not guaranteed or endorsed by the publisher.
